# Radiosurgical Septostomy: A Technical Report of Two Cases

**DOI:** 10.7759/cureus.94495

**Published:** 2025-10-13

**Authors:** Guillermo Axayacalt Gutierrez-Aceves, Miguel Angel Celis-Lopez, Sergio Moreno-Jimenez, Jose Alfredo Herrera-Gonzalez, Alejandro Rodriguez-Camacho, Jesus Alejandro Rodriguez-Torres

**Affiliations:** 1 Radioneurosurgery Department, Instituto Nacional de Neurología y Neurocirugía “Manuel Velasco Suárez”, Mexico City, MEX; 2 Radioneurosurgery Department, Instituto Nacional de Neurologia y Neurocirugia “Manuel Velasco Suárez”, Mexico City, MEX; 3 Radioneurosurgery Department, Instituto Nacional de Neurologia y Neurocirugia “Manuel Velasco Suárez”, México City, MEX; 4 Academic Unit of Human Medicine and Health Sciences, Universidad Autónoma de Zacatecas “Francisco García Salinas”, Zacatecas, MEX

**Keywords:** asymmetrical hydrocephalus, linac-based radiosurgery, linear accelerator (linac), radiosurgery, radiosurgical septostomy, septostomy

## Abstract

Septostomy is a surgical procedure in which a hole or fenestration is created in the septum pellucidum, a thin membrane between the brain’s lateral ventricles. Here, we describe radiosurgical septostomy as an alternative to the treatment of asymmetric hydrocephalus. We report the cases of two males, 46 years old and 64 years old, diagnosed with meningioma and asymmetric hydrocephalus. Brain MRI and cranial CT showed a left lateral hydrocephalus in both cases. Both patients underwent a radiosurgical septostomy on the TrueBeam STX platform. The target used was the septum pellucidum on its avascular portion. The prescribed doses were 120 Gy and 100 Gy, respectively. After radiosurgical septostomy, symptomatic and imaging improvement was noted. MRI showed a reduction in ventricular dilation over a long follow-up period. There were no treatment failures or complications with the procedure. Radiosurgical septostomy is a minimally invasive, highly precise, secure, and effective treatment modality.

## Introduction

Septostomy is a surgical procedure in which a fenestration is created in the septum pellucidum, a thin membrane between the brain’s lateral ventricles. This procedure is typically performed endoscopically to address obstructive hydrocephalus, intraventricular tumors, or other pathologies that impede cerebrospinal fluid (CSF) circulation through the foramen of Monro [1]. The goal of septostomy is to restore the flow of CSF by creating a passage through the septum pellucidum, thereby alleviating the pressure caused by the obstruction [2]. Septostomy is considered a safe and reliable procedure when performed by experienced neurosurgeons, and it has shown high success rates in restoring CSF circulation with low complication rates in highly selected patients [3].

Septostomy is indicated in cases where the CSF circulation at the level of the foramen of Monro is obstructed due to various causes, such as intraventricular tumors; multiloculated cystic hydrocephalus; septum pellucidum cysts; isolated, membranous, or inflammatory lateral ventricles; and giant aneurysms [1]. Septostomy is typically performed endoscopically and can be considered a safe and successful treatment option for many causes of CSF circulation obstruction in the foramen of Monro. It has a high success rate in restoring CSF circulation and a low complication rate [4]. Septostomy is often performed in conjunction with other endoscopic procedures, such as endoscopic third ventriculostomy or tumor biopsy, and may be preferred over ventriculoperitoneal shunting in specific instances [5].

However, neuroendoscopic septostomy is a procedure that is not exempt from risks [6], including intraoperative complications such as hemorrhage (10.2%), as well as post-surgical complications, such as neuroendocrine dysfunction (12.7%) or damage to structures next to the fornix or internal veins [3]. The time of the procedure is associated with an increased risk of complications [6].

We report the procedure and results of the first two image-guided (frameless) radiosurgical septostomies with LINAC TrueBEAM STx platform (Varian Medical Systems, Palo Alto, CA, USA), performed at the Instituto Nacional de Neurología y Neurocirugía “Manuel Velasco Suarez” from Mexico City. To our knowledge, there are no previous reports of this procedure being performed. Additionally, there is prior experience with published procedures such as radiosurgical third ventriculostomy for hydrocephalus and radiosurgical tumor treatment.

## Technical report

Case 1

A 46-year-old male had a medical history of two grade 2 meningioma resections, clinically with incomplete right upper motor neuron syndrome and residual tumor. Radiotherapy was performed on the residual meningioma (54 Gy in 30 fractions). In addition, due to asymmetric hydrocephalus and the rejection of surgical intervention, we administered a 120 Gy unique dose to the septum pellucidum.

Case 2

A 64-year-old male had a medical history of diabetes and hypertension and two meningioma resections, with the last one being incomplete and with a left parietal ventriculoperitoneal catheter placement due to hydrocephalus. Clinically, he had left hemiplegia. RT was performed for the residual meningioma (54 Gy in 27 fractions). In addition, due to asymmetric hydrocephalus and the rejection of surgical intervention, we administered a 100 Gy unique dose to the septum pellucidum.

Technique, image, and fusion

With a 3.0 T magnetic resonator (General Electric (GE) Signa Twin Excite MRI Scanner, GE Medical Systems, Milwaukee, WI, USA), pre-treatment with MRI was performed with a volumetric weighted sequence (SPGR) in single T1, contrasted T1, and T2, all with 1 mm thick slices, a matrix size of 512 × 512, and a pixel size of 0.45 mm without separation. For the fixation system, we opted for a three-component thermoplastic mask for radiosurgery (BrainLAB, Heimstetten, Germany) with anatomical neck support for noninvasive skull immobilization, fixed employing the universal base for the tomography table. Subsequently, a single skull simulation CT image was acquired using Siemens SOMATON Sensation 64 CT (Siemens Medical Solutions, Malvern, PA, USA) with 0.7 mm thick slices, matrix reconstruction size 512 × 512, and no gaps. Both images were fused using Brainlab Elements Image Fusion (version 3.0.1.6), which uses the registration method of anatomical matching (iterative) to match anatomical structures present to obtain accurate anatomical geometric information for the target treatment.

Definition of target volume and organs at risk

To define the target structure and delimit the organs at risk (OARs), the area of the septum was sought anatomically and away from the foramen of Monro to avoid damage to the fornix. Thus, the area to be treated was defined as 5-10 mm posterior to the foramen of Monro, halfway between the corpus callosum and the fornix.

Prescription dose, dose distribution, and treatment planning

The prescription dose to the isocenter was 120 Gy and 100 Gy for the two patients, respectively. Multiple non-coplanar arcs were used with a 6 mm BrainLab SRS cone. The BrainLAB iPlan RT Dose system (Version 4.5.5) was used to plan treatment with cone collimation, which uses the Clarkson dose algorithm for the calculation (Table 1).

**Table 1:** Treatment plan characteristics and dose volume calculations of both cases.

Parameter	Case 1	Case 2
Dose (Gy)	120	100
Cone (mm)	6	6
Energy	6 FFF	6 FFF
Calculation algorithm	iPlan/Clarkson	iPlan/Clarkson
Calculation matrix (mm)	0.7 × 0.7 × 0.7	0.7 × 0.7 × 0.7
CT slice thickness (mm)	0.7	0.7
Number of arcs	30	26
Fixation	BrainLab/Frameless	BrainLab/ Frameless
Localization	EXACTRAC	EXACTRAC
Position correction	EXACTRAC	EXACTRAC
Dose volume histogram		
Brain V12 (ccm)	3.24	2.32
Dmax (Gy) lens	0.365	0.71
Dmax (Gy) visual path	0.554	0.5
Dmax (Gy) brainstem	0.217	2.68
Dmax (Gy) cochlea	0.087	0.07
Dmax (Gy) pituitary	0.083	-

The geometric irradiation consisted of 15 (120 Gy) and 13 (100 Gy) couch movements in clockwise (CW) and counterclockwise (CCW) gantry rotation; hence, the total number of arcs was 30 (Table 2) and 26 (Table 3), respectively.

**Table 2:** Arch setup of Case 1.

Arc length (IEC61217)		Rtn table
350	190	0
190	350	0
170	10	0
10	170	0
170	10	325
10	170	325
170	10	330
10	170	330
170	10	335
10	170	335
170	10	340
10	170	340
170	10	345
10	170	345
170	10	350
10	170	350
170	10	355
10	170	355
350	190	5
190	350	5
350	190	10
190	350	10
350	190	15
190	350	15
350	190	20
190	350	20
350	190	25
190	350	25
350	190	30
190	350	30

**Table 3:** Arch setup of Case 2.

Arc length (IEC61217)		Rtn table
320	210	0
210	320	0
140	30	325
30	140	325
140	30	330
30	140	330
140	30	335
30	140	340
140	30	345
90	170	350
320	190	5
190	320	5
320	190	10
190	320	10
350	210	15
210	350	15
350	210	20
210	350	20
350	210	25
210	350	25
350	210	30
210	350	30
350	210	35
210	350	35
330	220	37
220	330	37

**Figure 1:**
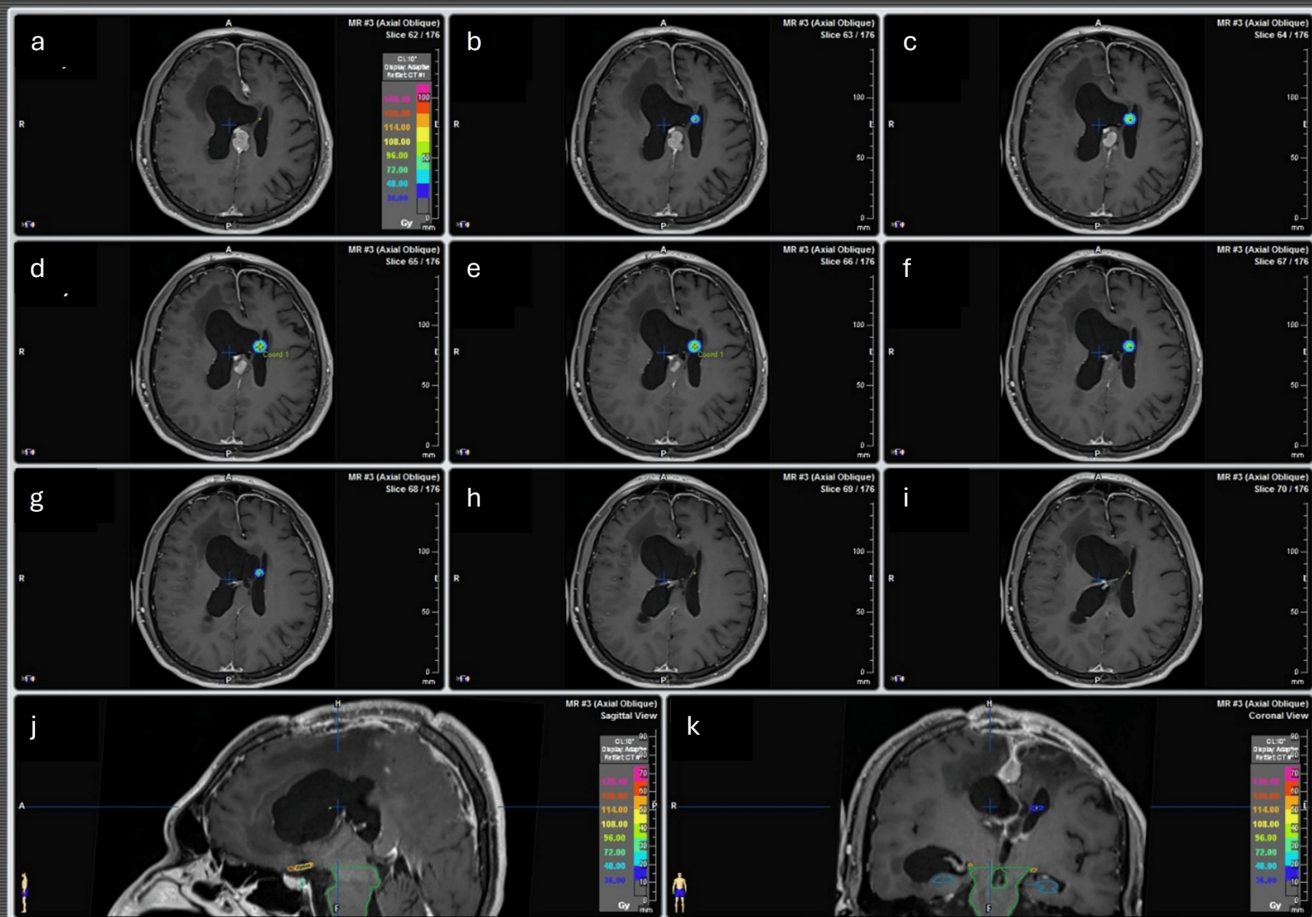
Dose distribution of Case 1. (a-i) Dose distribution on different axial slices. (j) Dose distribution on the sagittal slice. (k) Dose distribution on the coronal slice.

**Figure 2:**
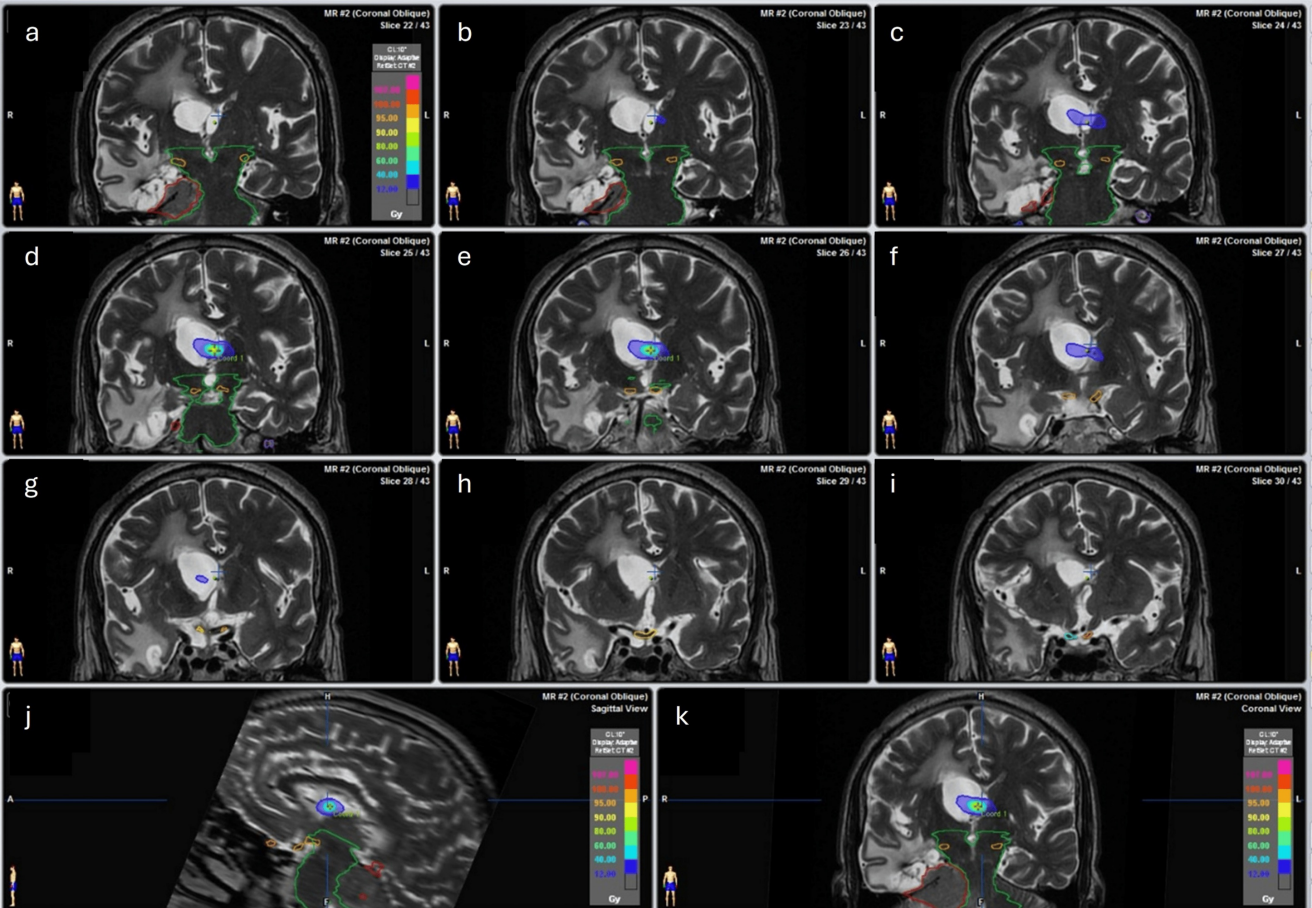
Dose distribution of Case 2. (a-i) Dose distribution on different axial slices. (j) Dose distribution on the sagittal slice. (k) Dose distribution on the coronal slice.

**Figure 3:**
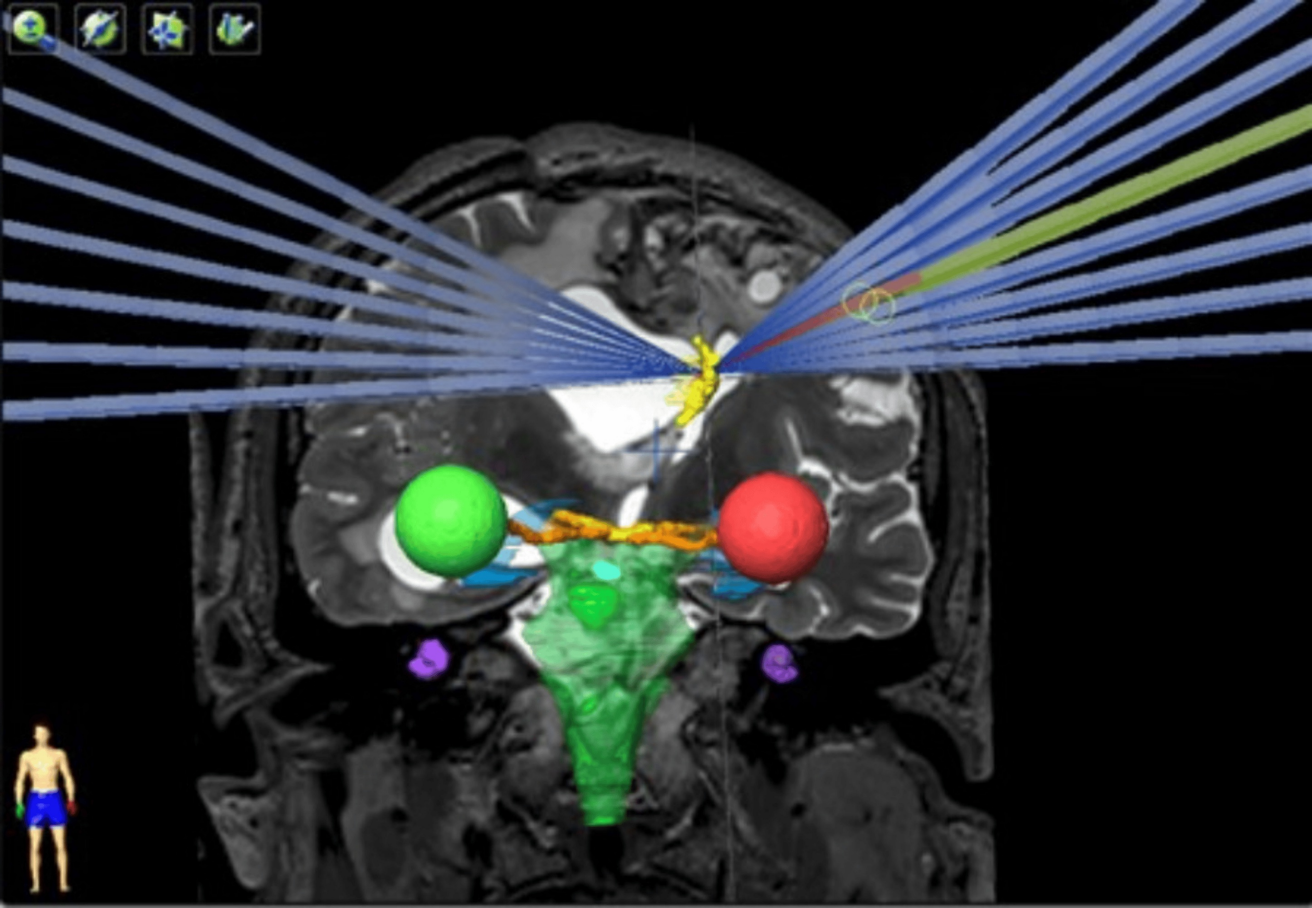
Treatment plan geometry of Case 1.

**Figure 4:**
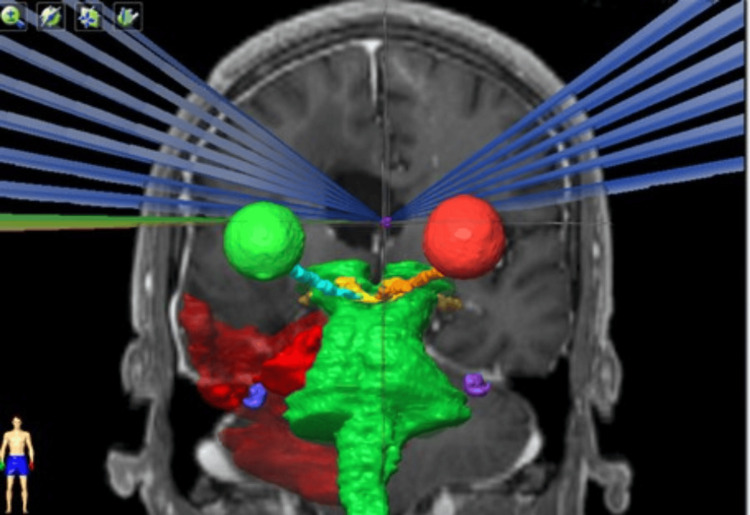
Treatment plan geometry of Case 2.

The conformity dose used (Figures 1, 2) and the dose distribution were elongated on the left-right axis (lateral ventricle enlarged). This was done to reduce dose to the normal tissue, because most of the dose is deposited inside the ventricles, and, on the other hand, the target that consists of a thin layer of tissue is crossed by the longest dose profile, which escalates the probability of appropriate dose coverage of the septum without normal tissue compromise (Figures 3, 4).

Treatment delivery

Both patients were treated in a LINAC TrueBEAM STx, with 6X-FFF energy (without flattening filter), with a dose rate of 1,200 MU/minute. The treatment lasted 72 minutes and 55 minutes, respectively. Patient immobilization was performed with a BrainLab mask for frameless image-guided radiosurgery. Localization is also performed using the BrainLab ExacTrac (ET) 6.2 system. Image guidance integrates an optical positioning system based on infrared, a three-dimensional (3D) radiographic positioning system consisting of the acquisition of two oblique images, kV X-ray stereoscopic (X-ray 6D), and a robotic couch that can move in six degrees of freedom for patient position correction. The verification of the initial position and each table movement was performed with the coregistration of the images acquired by the ET and the 3D image data of the simulation CT.

Patient quality assurance and end-to-end test

**Figure 5:**
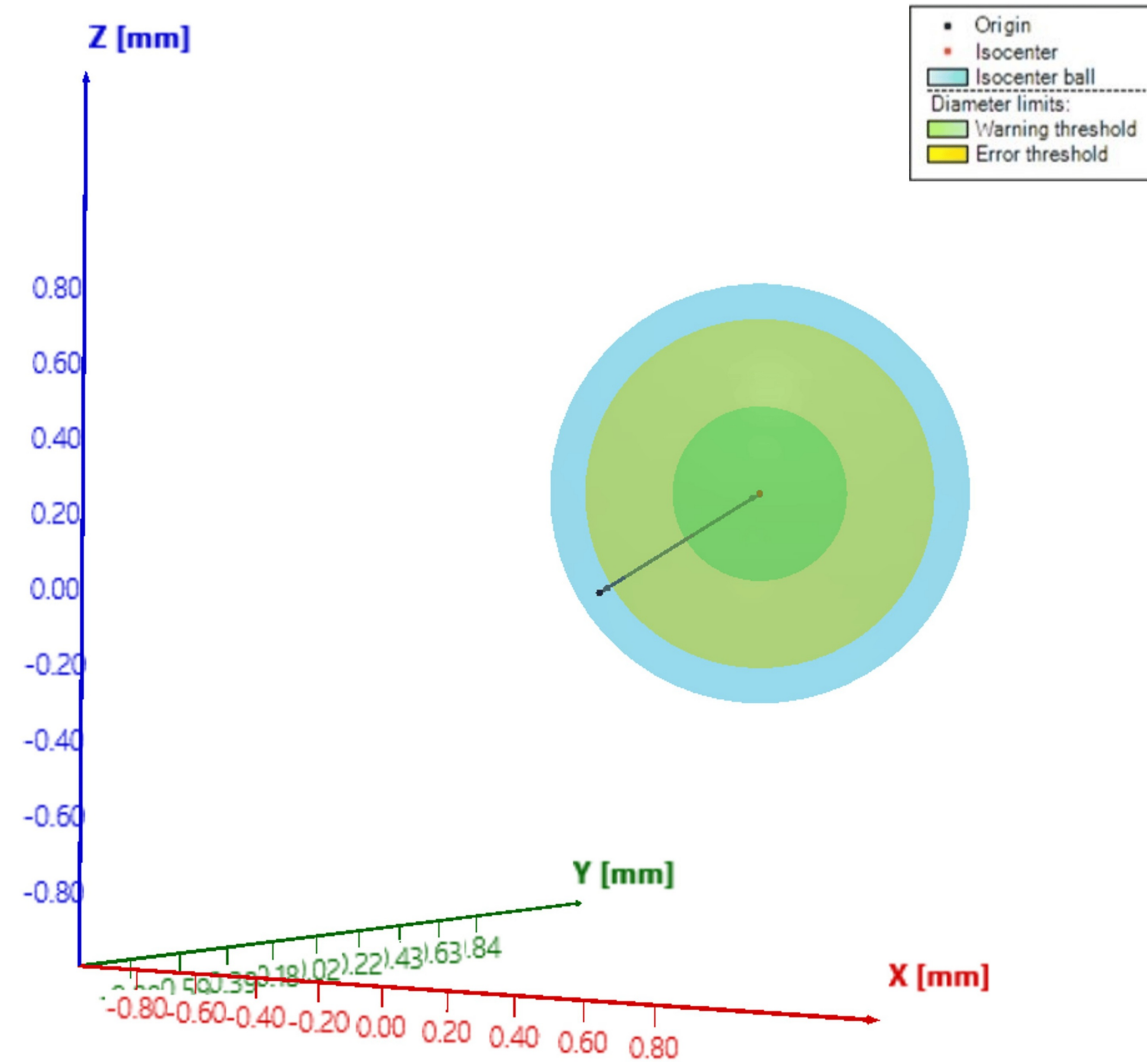
Winston-Lutz (WL) test results. The image shows Isoball WL test results with PTW® software analysis.

The Winston-Lutz (WL) test is routinely done using the Brainlab WL phantom in two distinct methodologies, with radiochromic film and the portal vision system. The results of these WL tests are below 1 mm on average, applying couch rotation corrections. Another WL test is executed with the PTW Isoball phantom. This system allows for measuring the LINAC error, localization error, and treatment error according to the original WL test concepts. The phantom is aligned with the linac lasers, and then several images with the linac IGRT image system are acquired independently for collimator, gantry, and couch (Figure 5).

**Figure 6:**
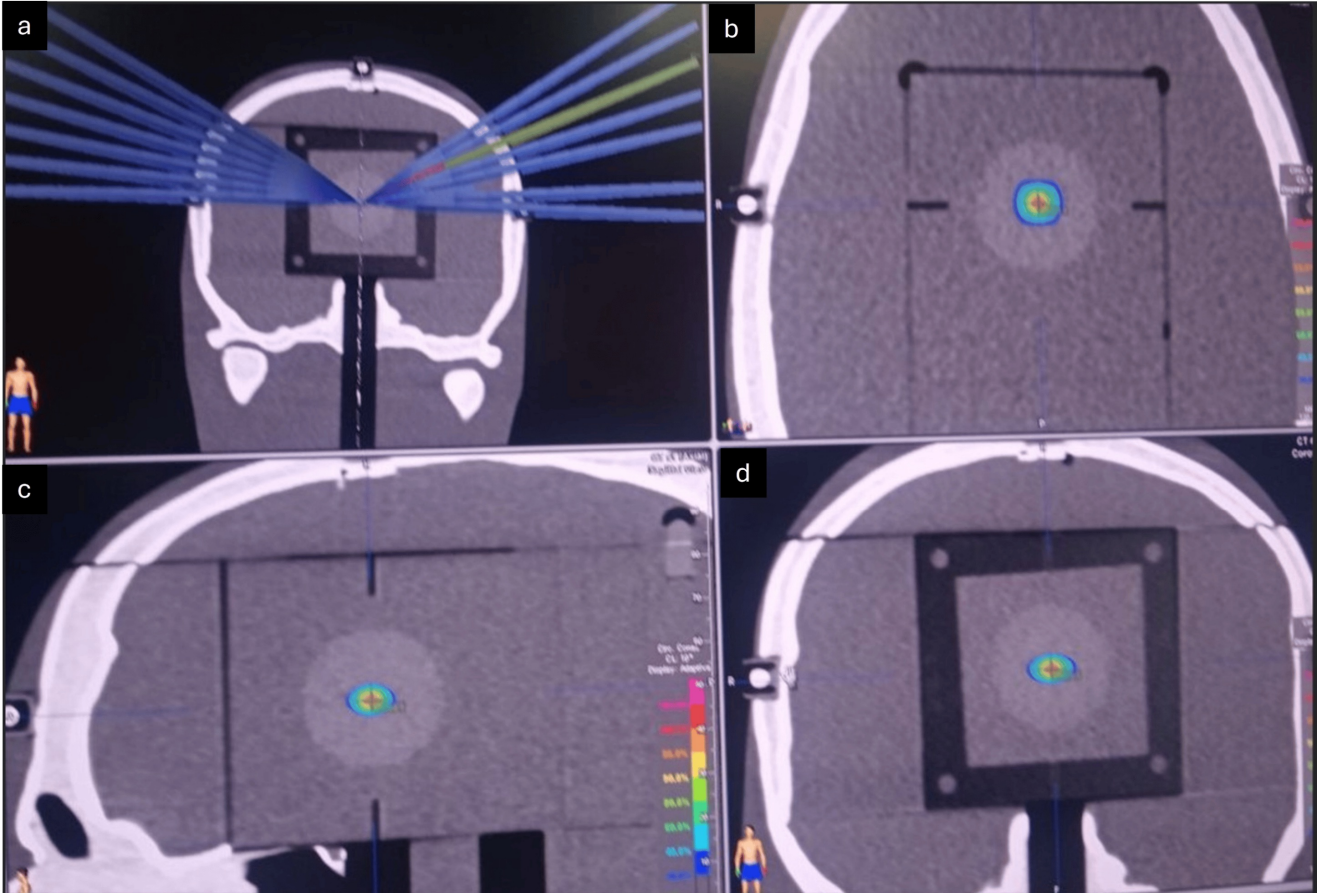
Dosimetric film quality assurance using STEEV Phantom. The a) shows the dosimetric film with the arches on the coronal CT scan slice, b) shows the dosimetric film on the axial slice, c) shows the dosimetric film on the sagittal slice, and d) shows the dosimetric film on the coronal slice.

**Figure 7:**
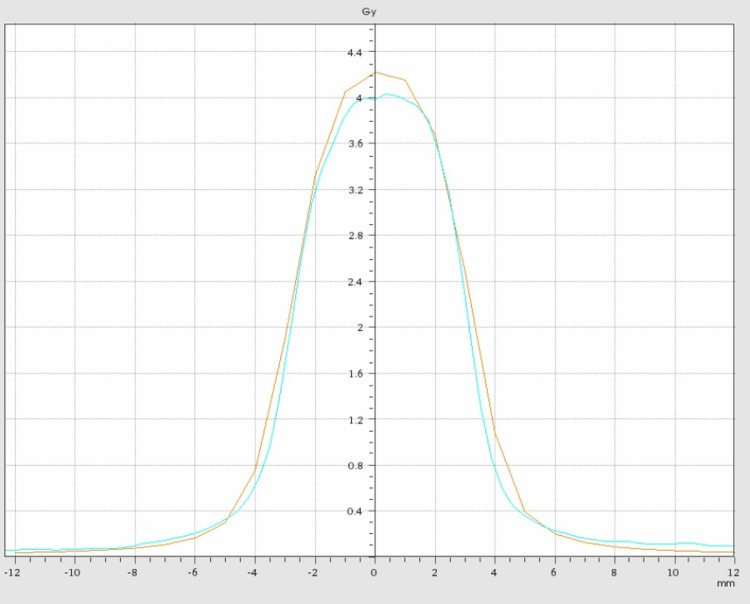
Comparison of dosimetric film profile between the calculated and measured dose.

For patient quality assurance (QA), an end-to-end test was performed using the STEEVE® anthropomorphic phantom (CIRS). This phantom has different material densities to take into account radiation transport across bone, cerebrum equivalent tissue, and others. The phantom goes through the entire treatment process, from CT simulation, planning, and treatment delivery. Radiochromic film EBT4 was used for dosimetric measurements (Figures 6, 7).

Results

Postprocedure Course

**Figure 8:**
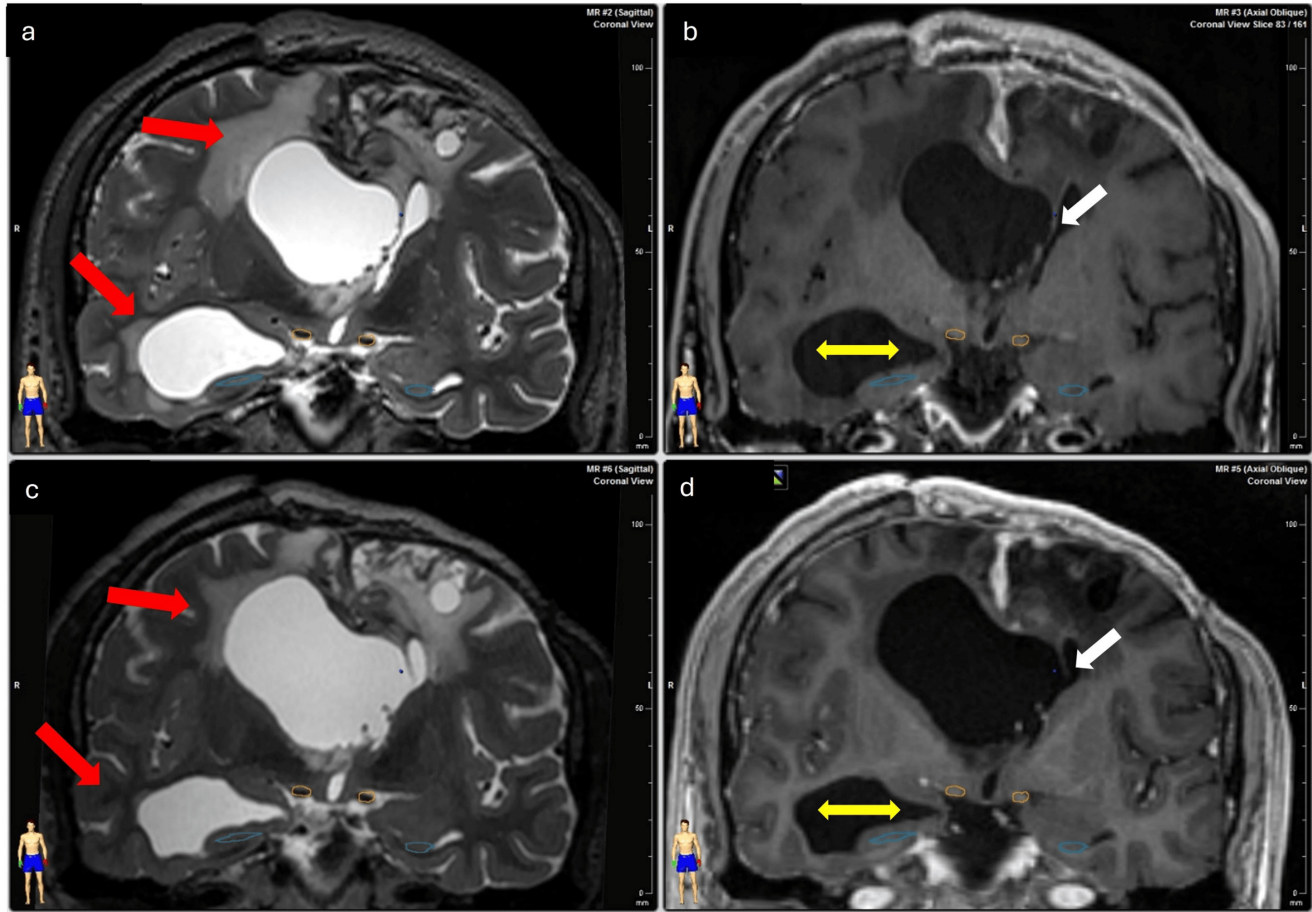
Post-treatment changes (Case 1). (a, b) Treatment images in coronal sections, obtained in T2 and T1 sequences, respectively. (c, d) Six months after treatment in coronal sections, obtained in T2 and T1 sequences, respectively. The red arrows show the improvement of the transependymal migration, the yellow arrows show the decrease in ventricular volume, and the white arrows show the fenestration of the septum pellucidum.

The initial follow-up was conducted through a clinical evaluation, which included an extensive neurological examination. The initial symptoms were decreased alertness, headache, and decreased strength, which improved after treatment (improved alertness, decreased headache, and increased strength), during which a clear improvement was observed in both patients. Likewise, CT scans were performed at five days and one month, followed by MRI every six months, and annually to verify the results by imaging (Figure 8).

**Figure 9:**
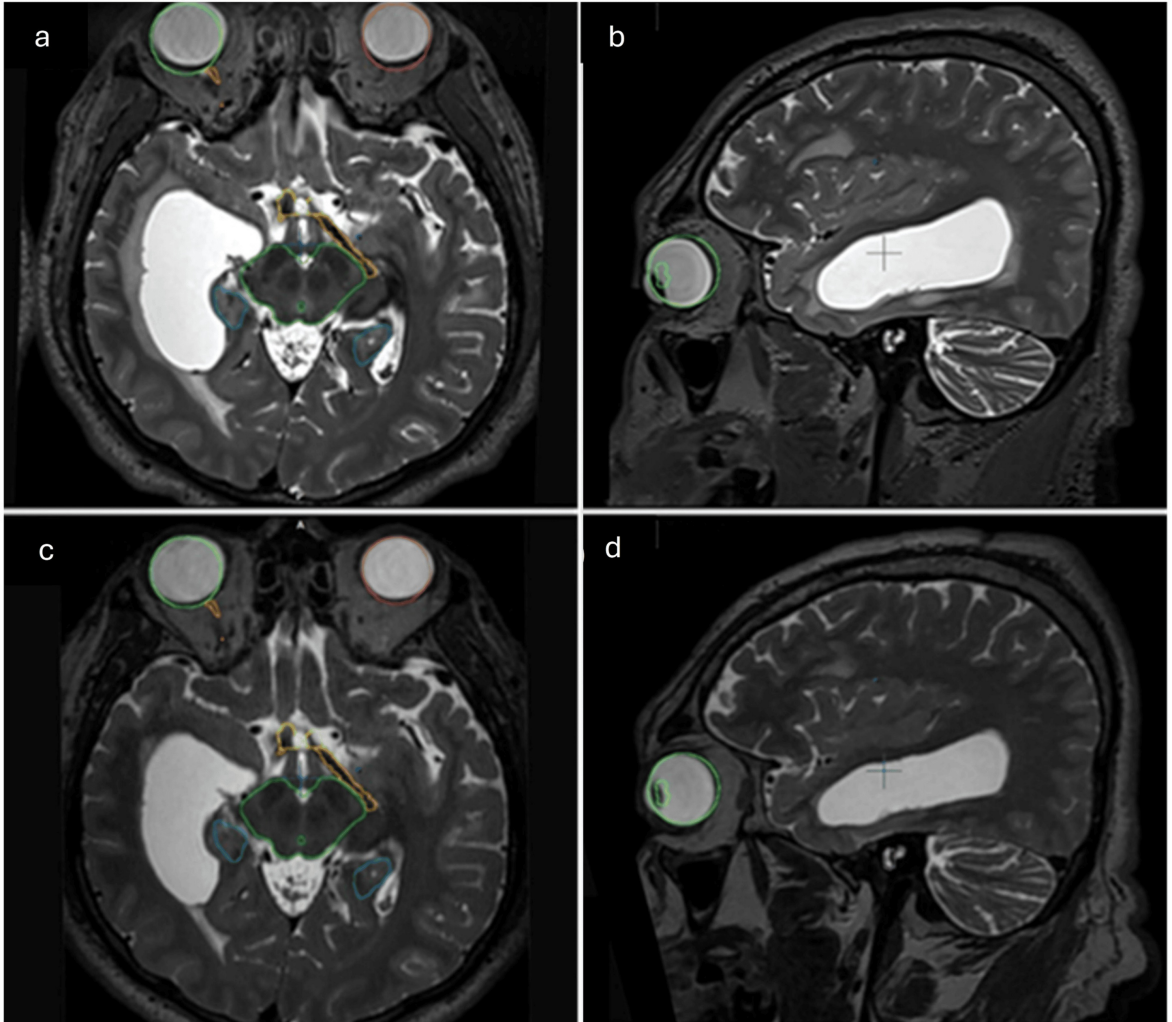
Follow-up images (Case 1). The image shows hydrocephalus and transependymal migration reduction on different slices of Case 1. The upper images show the case before the treatment, and the lower images show the case after one year of the treatment. (a, c) Axial slices. (b, d) Sagittal slices.

**Figure 10:**
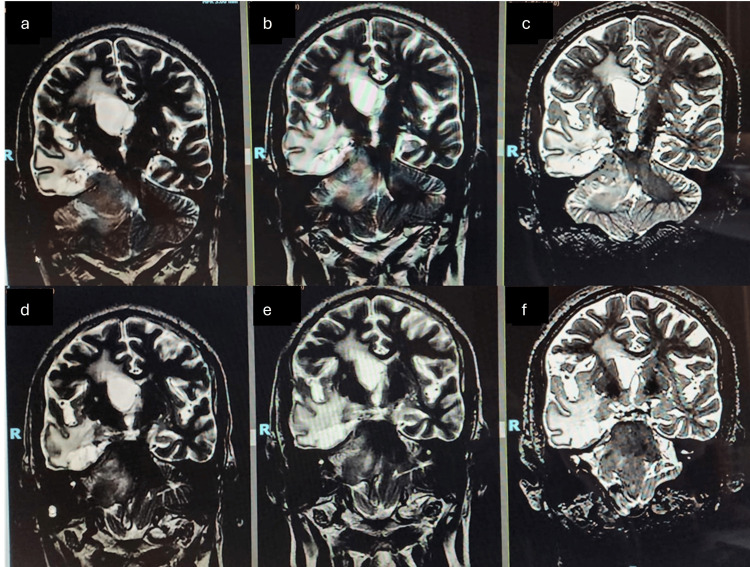
Follow-up images (Case 2). A reduction in hydrocephalus on different slices of Case 2. (a, d) Pretreatment images. (b, e) Images obtained after a year of treatment. (c, f) Images after two years of treatment.

No steroid medication was necessary for the patients, requiring only analgesics, such as paracetamol, at effective doses for one week. The patients’ improved alertness and symptoms, such as headaches (before treatment, 9/10 on the Visual Analog Scale, and after treatment, 4/10 on the Visual Analog Scale), decreased during the first 48 hours post-treatment. MRI T1, T1 contrast, T2, and fluid-attenuated inversion recovery imaging sequences were obtained, confirming the procedure’s success due to an improvement in the dimensions of the lateral ventricles and the periventricular edema (Figures 9, 10).

Regarding meningiomas, both patients continued with the protocol follow-up in a multidisciplinary manner; however, due to their histological nature, they were characterized as difficult to control and are currently under symptomatic treatment and surveillance.

During treatment and follow-up, no data on alterations secondary to radioneurosurgery were obtained, and the patients’ condition was always consistent with the documented tumor lesion, but not with the hydrocephalus.

In the short term, we observed clinical improvement within the first 48 hours post-procedure, with no subsequent clinical regression. At five days post-procedure, the septum’s fenestration was noted. During follow-up, we observed a reduction in ventricular volume and size and an improvement in transependymal migration. The two-year and one-year follow-ups are shown in Table 4 and Table 5, respectively.

**Table 4:** Follow-up of ventricular volume in Case 1.

Ventricular volume	Pre-treatment	One-year follow-up
Total volume	93.5 cc	90.4 cc
Right ventricle volume	63.9 cc	56.1 cc
Left ventricle volume	29.6 cc	34.3 cc

**Table 5:** Follow-up of ventricular volume in Case 2.

Ventricular volume	Pre-treatment	Two-year follow-up
Total volume	12.5 cc	11.7 cc
Right ventricle volume	11.8 cc	9.3 cc
Left ventricle volume	0.7 cc	2.4 cc

## Discussion

Currently, there are treatment procedures that are on the rise, such as endoscopic fenestration, which is the most common procedure (70.9%) [8]. Endoscopic procedures have become a valuable and frequently applied technique [9]. Neuroendoscopic septostomy improves the management of complex hydrocephalus by allowing the flow of CSF between ventricular compartments [6]. Considering the neural and vascular architecture, it must be carefully performed to avoid significant complications [10]. Careful evaluation is required to prevent damage to critical structures such as the fornix during surgery [11], with imaging results showing hydrocephalus resolution and ventricular dilation reduction [12]. These procedures are performed under general anesthesia and neuronavigation with intraoperative guidance [9]. Minimally invasive techniques have excellent treatment efficacy, with the possibility of obtaining a biopsy if necessary [13]. The perforation should be performed above the upper border of the fornix and dorsal to the interventricular foramen to minimize the risk of damage to the septal nuclei and precommissural fibers [10]. Endoscopic septostomy should only be performed when the anatomical landmarks are identified and it is possible without significant surgical risk [9].

Stereotactic radiosurgery has been well-accepted as a therapeutic modality in various types of neurological diseases, such as primary and secondary malignant tumors, benign tumors, arteriovenous malformations, and even some functional procedures, such as trigeminal neuralgia, thalamotomies, pallidotomies, cingulotomies, and callosotomies [14-20].

Several studies have been published in the literature describing stereotactic radiosurgery procedures to alleviate functional disorders, using doses between 150 and 200 Gy in highly eloquent areas such as the thalamus, globus pallidus, pituitary gland, and subthalamus [15,16,18,19]. In our center, we have experience using 100-120 Gy for hydrocephalus treatment, i.e., radiosurgical third ventriculostomy. With this dose, we have achieved good results, likely because in hydrocephalus, these structures are submitted to hydrostatic pressure, and this force can help the ventricular system communicate in the short term: the third ventricle floor to the basal cisternal system, the septum pellucidum to the lateral interventricular flow, and the lamina terminalis to the subarachnoid space [7].

On follow-up, we found clinical improvement during the first 48 hours post-treatment. Changes in the ventricular size are usually challenging to find, even after weeks or months. The clinical follow-up is the main indicator of response in the short term. The fenestration in the septum pellucidum after the procedure is difficult to determine because the thickness of the fenestrated structure complicates its immediate observation by imaging. In addition, it is firmly documented that the ventricular volume does not change in the short term [7].

In our cases, we found sustained clinical improvement in the symptomatology during the follow-up, and the imaging follow-up showed progressive improvement in the ventricular size and the periventricular edema. In one patient, we observed a “hole” in the septum pellucidum five days post-procedure. Otherwise, we did not find changes in the obstruction of the foramen of Monro. We did not find any complications secondary to the radiosurgery treatment.

This method of treating asymmetrical hydrocephalus is safe and effective; however, it is not yet an alternative to endoscopic septostomy, as more patients and larger studies are needed to confirm its efficacy. This technique can be used to treat the brain tumor and the asymmetric hydrocephalus with irradiation, optimize time and resources, and avoid the surgical and anesthetic risks.

## Conclusions

Radiosurgical septostomy is an alternative procedure that avoids the potential complications associated with traditional surgery. It does not require hospitalization or the use of steroids and has minimal side effects. Additionally, this method offers the benefit of treating two conditions simultaneously, thereby optimizing both resources and time. Radiosurgical septostomy is a minimally invasive, precise, and safe procedure. We consider it to be a viable option for highly selected patients with asymmetric hydrocephalus. More patients and larger studies are needed to confirm its efficacy.
